# Robotic support for older adults with cognitive and mobility impairments

**DOI:** 10.3389/frobt.2025.1545733

**Published:** 2025-04-07

**Authors:** Samuel A. Olatunji, James S. Shim, Adam Syed, Yao-Lin Tsai, April E. Pereira, Harshal P. Mahajan, Raksha A. Mudar, Wendy A. Rogers

**Affiliations:** College of Applied Health Sciences, University of Illinois Urbana-Champaign, Champaign, IL, United States

**Keywords:** aging, human-robot interaction, physical disability, cognitive impairment, technology acceptance

## Abstract

**Introduction:**

Robots have the potential to support older adults with cognitive impairments and mobility impairments in daily tasks that could promote their independence, enhance their abilities, ensure safety, and lower healthcare costs.

**Method:**

Using a participatory design approach, we focused on identifying the functional capabilities of the Stretch robot to support older adults with various cognitive or mobility impairments. Twelve participants (aged 60–97) were recruited to interact with the robot and give feedback regarding support in a home environment. Stretch is a mobile robot manipulator designed to support everyday activities using a lightweight telescoping arm mounted on a mobile base. We conducted a semi-structured interview with participants as they observed and interacted with Stretch, performing tasks such as providing reminders, picking up and delivering items, and facilitating video calls.

**Results and Discussion:**

The participants were asked to share potential areas of application related to their daily activities to illustrate how Stretch could support them in their homes. Our user-centered design approach provided a unique opportunity to understand the needs of older adults with mobility impairments and cognitive impairments, to identify the type of tasks the robot could support, and to gain insights into potential facilitators and barriers for robot adoption.

## 1 Introduction

### 1.1 Aging and challenges in everyday living

The global population of older adults is rapidly increasing, reflecting a significant demographic shift that is reshaping societies around the world. In 2022, there were 771 million people aged 65+ years globally, accounting for almost 10% of the world’s population. It is projected that by 2030, 1 in 6 people in the world will be aged 60 years and over (World Health Organization, 2017). The increase in lifespan, linked to remarkable breakthroughs in healthcare and living conditions is an incredible societal achievement, but it also presents challenges to the healthcare systems, families, caregivers, and older adults (World Health Organization, 2017). Due to normative age-related changes and health conditions commonly associated with aging, many older adults encounter difficulties in performing their daily activities, which can affect their independence. They face challenges such as social isolation, loneliness, and difficulties engaging in meaningful activities. Additionally, some experience further challenges due to cognitive or physical impairments.

Estimates suggest that over 35% of people age 65+ in the United States live with a cognitive or physical impairment (Rajan et al., 2021; Dhana et al., 2022). Mild cognitive impairment (MCI) is one of the most common forms of impairment and refers to impairment in one or more cognitive domains without fulfilling the diagnostic criteria for dementia (Roberts and Knopman, 2013). Approximately 12%–18% of people aged 60 or older are living with MCI (Rajan et al., 2021). Persons with MCI experience deficits in complex instrumental activities of daily living (IADLs) managing finances and health (e.g., managing medications) (Jekel et al., 2015). Older adults with physical impairment characterized by upper and/or lower body mobility disabilities face challenges with IADLs which limits their autonomy (Freedman and Spillman, 2014). Furthermore, common co-morbid health conditions such as visual and auditory impairments, hypertension, obesity, pain can exacerbate challenges faced by both individuals with cognitive and mobility impairments. Many of these older adults require assistance with cognitive tasks (like receiving reminders, guidance on task details, or cues for procedures) and physical activities (such as delivering an item or picking up). Support personnel may not always be available or accessible to assist with these tasks (Koon et al., 2020). Technologies such as assistive robots have the potential to fill that gap.

### 1.2 Role of technology

Technology provides a resource for addressing the challenges of an aging population by providing innovative assistive services, enhancing health, wellbeing, and safety, and creating opportunities for engagement (Czaja et al., 2019). In this context, assistive robots could play a vital role in supporting older adults aging in place successfully. A growing array of robots, either commercially available or in the development phase, are designed to assist older adults (Ostrowski et al., 2019) thereby enhancing quality of life. These robots offer multifaceted support, catering to physical, cognitive, social and safety needs. For instance, robots are designed to assist with household chores provide (Beer et al., 2012), offer cognitive stimulation (e.g., robots supporting MCI patients, Law et al., 2019), support social communication (e.g., social companion robots, Wada et al., 2004), and perform safety monitoring (e.g., robots supporting wellness check robots, Smarr et al., 2011).

Robots have the potential to support older adults with cognitive or mobility impairments in daily tasks (Olatunji et al., 2024; Lukasik et al., 2021; Costanzo et al., 2024; Granata et al., 2013; Sawik et al., 2023). This support could promote their independence, enhance their abilities, ensure safety, and reduce healthcare costs associated with hiring caregivers, medical consultations, or monitoring (Pino et al., 2015; Korchut et al., 2017; Dosso et al., 2023; Zhao et al., 2023). However, the acceptance of robotic support is influenced by the usability of the robot within the specific context of the target users’ needs. There are unmet needs of the target users that assistive robots could support as priority tasks. A key gap remains in matching the robot’s capabilities to support these priorities. To address this gap, we used a participatory design approach to assess the feasibility of using a robot to support older adults with mobility and/or cognitive impairments.

### 1.3 Aim and research questions

We aimed to identify the functional capabilities of the robot aspired by older adults with a range of cognitive and mobility impairments through the following research questions:

RQ1: What capabilities should a robot have to support older adults with home tasks?
RQ2: What kind of tasks and activities would the older adults want the mobile manipulator robot to support?
RQ3: What are the facilitators and barriers to robot adoption by older adults?

To address these questions, we implemented a participatory design study wherein an assistive robot was embedded in a home simulation environment to support older adults with various activities of daily living. Quantitative and qualitative evaluations were conducted to explore the utility and benefits of using a robot to support the older adults and their attitudes towards the robot potentially assisting them during everyday activities in their own home environment.

A key aspect of participatory design is engaging target users in the design process. However, ensuring that users are comfortable with the technology is critical to eliciting meaningful feedback using these methods. This is especially true for newer technologies, such as assistive robots for older adults. Lack of familiarity or discomfort with robots could impact their attitude towards it. Hence, in this study, we systematically captured user perceptions and gathered feedback by gradually increasing participant’s exposure to the robot. This approach increased their comfort in the situation and facilitated obtaining valuable insights regarding robot design.

## 2 Materials and methods

### 2.1 Participants

A total of twelve participants 60 years or older (M_age_ = 73.4, SD_age_ = 9.08, Female = 8), fluent in English with normal or corrected vision and hearing (i.e., able to see and hear instructions) were recruited. Potential participants were identified through outreach to relevant community organizations, senior living facilities, and other public spaces. Participants were also recruited through email distribution, e-flyers on social media, and local aging resource communities. The Rehabilitation and Engineering Research Center on Technologies to Support Aging-in-Place for People with Long-Term Disabilities participant registry was also used for recruitment. The researchers’ contacts also supported using snowball recruiting to attract a variety of older adults. Upon expressing interest, participants were contacted by members of the research team to initiate the screening process.

Participants with a range of cognitive and mobility abilities were recruited, resulting in four groups: (1) cognitive impairment plus mobility impairment; (2) cognitive impairment only; (3) mobility impairment only; and (4) no cognitive or mobility impairment. To be considered as having a cognitive impairment, participants had to score between 22 and 37 on the Modified Telephone Interview for Cognitive Status (TICS-M; Cook et al., 2009) and below or equal to 26 on the Montreal Cognitive Assessment (MoCA; Nasreddine et al., 2005). For mobility impairment, criteria included having serious difficulty in walking or climbing stairs (or inability to do so), difficulty raising a 2-L bottle from waist to eye level, or difficulty using hands and fingers for prehension tasks for at least 10 years. Of the twelve participants, three had both cognitive and mobility impairment; four had cognitive impairment only; three had mobility impairment only; and two were without cognitive or mobility impairments. Exclusion criteria for both populations were having a diagnosis of Alzheimer’s or other dementia and/or a history of significant psychological illness.

### 2.2 Robot

**FIGURE 1:**
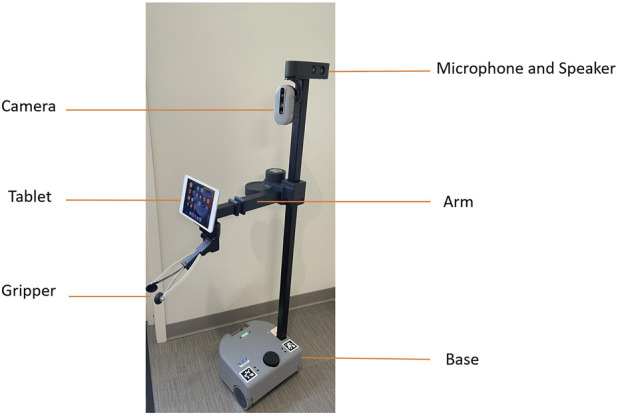
Stretch robot, research edition 2 (RE2) by hello robot (Hello Robot, 2021).

The robot used in the study was Stretch (research edition 2, developed by Hello Robot Inc.), which is a mobile robot manipulator designed to support everyday activities using a lightweight telescoping arm mounted on a mobile base (see Figure 1). The robot can be operated at two levels of autonomy: teleoperation level and semi-autonomous level. At the teleoperation level, the robot’s arm joints, and mobile base are directly controlled through the game controller. At the semi-autonomous level, the action sequences of the robot arm and base can be captured as a user-defined function through a web interface (Olatunji et al., 2024). Stretch is an assistive robot, meaning that it can give aid or support to human users (Rogers and Mitzner, 2017). The robot classification was defined by the robot’s function, not by the robot’s appearance.

We employed the teleoperation mode through the robot’s game controller to simulate voice control of the robot for potential tasks that the robot could be programmed to execute at a higher level of autonomy. This provided the opportunity to understand the perceptions of the older adults regarding tasks, behavior towards the robot, and overall impression of the interaction, with the assumption that the technology would work as expected. It also facilitated consistency in the robot’s actions for all participants.

### 2.3 Procedure

The study took place in the McKechnie Family LIFE Home at the University of Illinois Urbana-Champaign. The home simulation space is the LIFE Home is a full-scale and functional two-bedroom home space with an observation area, outfitted with cameras and speakers. We conducted this study as a one-time visit that lasted about two and a half hours. At the beginning of the session, participants provided informed consent. They completed the TechSAge Background Questionnaire (Remillard et al., 2020), which gathered information about age, race, ethnicity, general health information including current conditions, vision, hearing, and motor capabilities; the Robot Familiarity and Use Questionnaire, which assessed participants’ familiarity with robots in terms of hearing about, using, or operating them (Smarr et al., 2012); and the Mobile Device Proficiency Questionnaire (MDPQ-16; Roque and Boot, 2018) to evaluate their proficiency with mobile devices; and the Montreal Cognitive Assessment (MoCA), a global cognitive screener (Nasreddine et al., 2005). A baseline measure of trust to assess their willingness to trust a robot (Malle and Ullman, 2021) was administered at the start and again at the end of the session to assess changes in perception over the course of the study.

**FIGURE 2:**
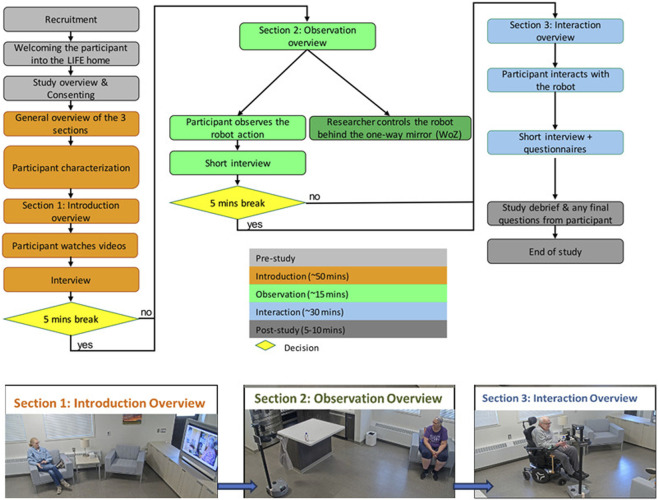
Protocol design and illustrations of the gradual introduction to Stretch.

The study consisted of three study sections: introduction, observation, and interaction with breaks offered in between. This systematic introduction of the robot allowed older adults to become more comfortable interacting with the robot and gave them additional time to provide their insights (see Figure 2).

The introductory section involved introducing the robot and its capabilities to the participant through watching video recordings of the robot’s activities. During this section, participants were encouraged to think aloud, to express their thoughts and impressions, perceptions of the robot on what it was doing and how it was doing what it was doing, and to share opinions about potential support from the robot. This was followed by a short interview to explore their overall perceptions of the robot. The entire introduction section lasted 30 min.

In the observation section, participants watched Stretch interact with a research team member to perform tasks such as providing reminders and picking up and delivering items in the home simulation area. Participants were prompted to think aloud and share their thoughts about the robot, the tasks it was performing, the interaction between the robot and the research team member, and their perceptions about the potential of the robot to interact with them in a similar or different ways. The observations section took around 30 min in total, including the demonstration, think aloud, processing of ideas, and prompting questions.

In the interaction section, participants engaged directly with the robot. They received a delivery of a bottle of water and participated in a video call session with a research team member through the tablet on the robot. During this session, participants were reminded to think aloud, share their thoughts as they interacted with the robot, and to express their feelings, fears, worries, concerns or excitement about their interaction experience. The interaction section lasted for 30 min. The interaction was followed up by a semi-structured interview about overall perceptions of the robot.

After the interview, participants completed the following questionnaires: the Robot Trust Questionnaire (Malle and Ullman, 2021); the System Usability Scale (SUS; Brooke, 1996), administered after the interaction session to measure participants perceptions of the robot’s usability; NASA Task Load Index (NASA-TLX; Hart, 2006), used to evaluate the subjective workload experienced by the participants; Perceived ease of use and perceived usefulness questionnaires (Davis, 1989); and a task rating to assess participants’ willingness to use Stretch for various activities of daily living. Lastly, participants were debriefed and compensated for their participation (Amazon e-code of $50). The study was approved by the Institutional Review Board of the University of Illinois Urbana-Champaign.

### 2.4 Analysis

The study was composed of both quantitative and qualitative data. The qualitative data in audio format were transcribed with Otter.ai software. Afterwards the research team edited the transcripts to ensure they matched the audio verbatim. The transcripts were segmented into analysis units, which were composed of the different aspects of participants’ perceptions of the robot. We used thematic coding to identify key topics and themes relevant to the research questions. We developed a coding scheme (see Supplemental Material) by reviewing a random sample of three transcripts and extracting key topics and common themes based on literature related to human-robot interaction (HRI) and the themes that emerged from the participants’ comments (Braun and Clarke, 2006). This was an iterative strategy whereby categories were assigned using existing themes from the HRI literature and new categories were added based on the emerging themes. Three coders were calibrated by conducting seven rounds of independent coding on the same randomly selected transcripts. Each round was followed by a discussion of discrepancies and revision to the coding definitions. The final round of reliability resulted in an average of 80% agreement among the three coders. The remaining transcripts were divided among the three coders to code independently.

## 3 Results

Participants’ characteristics are illustrated in Tables 1, 2. Nine of the 12 participants received a score of more than 30 (out of 40) on mobile device proficiency, pointing to proficiency with basic and advanced uses of smartphones and tablets for various functions (Table 2). This reflected a sample population with some familiarity and independence using mobile devices. However, 11 participants had never heard about, seen, or used any of the robot categories and examples provided in the questionnaire.

**TABLE 1:** Participant characteristics.

Measure	Mean (SD)	Number of participants (%)
Age	73.4 (9.08)	
Sex (Female)		8 (66.7)
Ethnic Group (Caucasian)		12 (100)
Education		
High school		2 (16.7)
Some College or Associate’s degree		1 (8.3)
Bachelor’s		4 (33.3)
Master’s		4 (33.3)
PhD		1 (8.3)
Marital Status		
Single		1 (8.3)
Married		8 (66.7)
Widowed		3 (25.0)
Living Situation		
Alone		3 (25.0)
With their partner		8 (66.7)
With their child		1 (8.3)
Quality of Life	6.00 (0.74)	
Montreal Cognitive Assessment (MoCA)*	25.50 (3.55)	

* The Montreal Cognitive Assessment (MoCA) test is scored out of 30 points, with a higher score indicating better cognitive function. A MoCA, score of 26 or below is often considered an indicator of cognitive impairment, which could include conditions like mild cognitive impairment or dementia.

**TABLE 2:** Cognitive and Mobility Status alongside Baseline Characteristics of each Participant.

Participant	Cognitive impairment	Mobility impairment	Age	Sex	MoCA (Max = 30)	MDPQ (Max = 80)	Robot familiarity (Max = 5)
1	✓	✓	76	F	23	36.0	2.85
2	✓	✓	66	F	23	39.5	2.08
3	✓	✓	77	M	23	31.5	1.92
4	✓	✕	97	F	19	8.0	1.92
5	✓	✕	73	F	23	36.5	2.38
6	✓	✕	76	M	24	31.5	2.69
7	✓	✕	75	F	26	40.0	2.77
8	✕	✓	70	F	30	35.5	2.46
9	✕	✓	67	F	30	28.5	2.69
10	✕	✓	77	M	27	19.0	2.69
11	✕	✕	62	M	30	40.0	3.00
12	✕	✕	65	F	27	39.5	2.54

MoCA, Montreal Cognitive Assessment; MDPQ, Mobile Device Proficiency Questionnaire.

### 3.1 Robot capabilities needed to support home tasks for older adults

**FIGURE 3:**
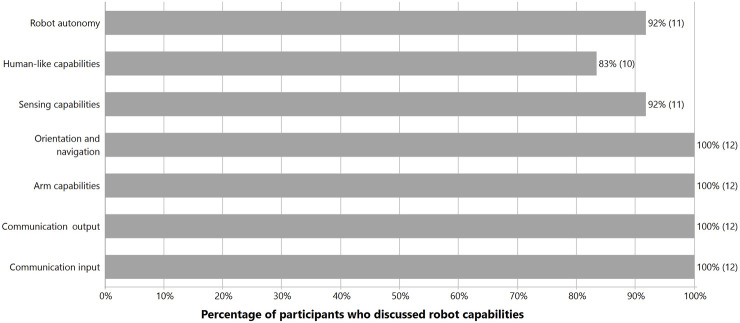
Robot capabilities mentioned by participants.

Participants shared their ideas about various capabilities they would expect an assistive robot to have if it would support them with home tasks. They commented on Stretch’s current capabilities while proposing ideas that would enable Stretch to better meet their needs. Their perceptions of the robot’s capabilities were analyzed, categorized into themes, and then tallied as seen in Figure 3. Details for each of these themes are provided in the subsequent subsections.

#### 3.1.1 Robot autonomy

We defined robot autonomy as Stretch’s capacity to decide its actions. Participants preferred the robot to have a level of autonomy that allows it carry out certain routine actions or scheduled tasks on its own such as cleaning, recharging itself, and scanning the environment for safety hazards. They wanted it to execute actions following specific voice commands. They wanted to be able to tell Stretch to deliver a bottle of water (or other items such as a book, or utensil in the kitchen) without it requiring their input to manually control it to get the item.

They wanted Stretch to autonomously know when it was wrong, and to know how to correct itself. They expected some level of intuitiveness from the robot to be able to make decisions based on what it senses but still give them, as the final deciding entity, the ability to overrule actions, veto actions, and halt undesired robot processes. Examples of comments are as follows:

*“If he can look through his film, his camera, and know that it’s wrong, or somehow know that it’s wrong himself.”* (Participant 3)

*“if it has the ability to learn if it makes a mistake, you cannot do it right the first time. Next time, it’s asked us to do the same thing can it learn and improve on that process, you know? That that would be something nice to see. Because I think if something does the same thing over and over again, and it’s failing every single time, you might look at that. Well, if it’s a kid that kid is going to learn.”* (Participant 9)

#### 3.1.2 Human-like capabilities

These were capabilities that the participants explicitly referred to as human-like capabilities they would like the robot to have (Note the use of pronouns in the previous quote from Participant 3). Most capabilities were expressed as human characteristics, behavior, or traits that they desired to see in the robot’s interactions with them. Examples of these include attributes like friendliness, politeness, intelligence, as seen in the following quotes:

*“… being a friend first. And then, like any real friend, like a human friend, they have a certain set of capabilities. Not every friend can do everything.”* (Participant 5)

*“Like a polite person … why should not it be a polite robot?”* (Participant 2)

*“very clever … following commands well.”* (Participant 6)

#### 3.1.3 Sensor capabilities

The robot was expected to have the capability to become aware of something via its sensors. Participants commented on the importance of the robot being able to distinguish between different items such as medication, items to pick up, furniture in the house so it does not collide with items within the home. They emphasized the importance of using these sensors when interacting with them (e.g., perceiving when they needed to get a rest or feeling stressed out or in the mood of a conversation). Example comments include:

*“Oh, yeah. Yes, I think it was. My only question was, when Stretch was handing the bottle to me, do I take it from ’em? Or wait for him to drop it? Or? Yeah, how do I, how do I get the cue of it’s now in my possession and not Stretch's possession? You know, I guess if I just pulled it or someone, gave it a Yeah, gave it a little pull. Stretch would sense it and release it.”* (Participant 10)

*“I would assume I mean, I cannot comprehend how Stretch would get the right pills? Maybe it needs to know what you’re taking. Identify that medicine, just double check the dispenser.”* (Participant 2)

#### 3.1.4 Orientation and navigation

Orientation and navigation are the capabilities of the robot to move around. Most of the participants appreciated the ability of the robot to navigate to most spaces, adapt to various floor types and move in different directions. The flexibility of navigation was a theme seen in most of the comments of the participants as they related this capability to the usefulness of the robot to help in different parts of the home, as illustrated by this example:

*“…the fact that it has so much flexibility to turn and hand things and move all kinds of directions, it’s really impressive”* (Participant 2)

However, some of the participants highlighted other orientation and navigation related expectations which could make it more adaptive in their homes. For example,

*“Needs to be able to swivel more. It needs to be able to go completely around.”* (Participant 5)

*“See how he can only move in one direction to get to me? That’s weird because he could have just turned around.”* (Participant 6)

*“I think it would be helpful to be faster.”* (Participant 5)

#### 3.1.5 Arm capabilities

The participants expressed the benefit of Stretch having an arm and dexterous wrist to pick up different items. Comments were related to the robot’s capability to pick up, deliver, arrange, retrieve, and place items correctly as desired:

*“An extra hand. It could be an extra hand. … I live alone. So there have been times when an extra pair of hands or an extra hand can come in handy”.* (Participant 9)

*“it could pick up anything and get it out of his way”* (Participant 3)

*“its already showing have it help with people whose cognitive impairment is greater than mine”* (Participant 11)

*“I think it'd be very useful, especially getting things up off the floor with something flatter and hard. … newspapers turning newspapers or something, turning something like that. Turning the pages of a book”* (Participant 9)

*“that’s very important for older people to run the can opener and opening bottles…*. *I’d like for him to hold my Recipes Cooking and pull them out for me.”*(Participant 8)

The participants’ comments reflect the perception of older adults concerning the degree of importance and criticalness of Stretch’s arm and gripper capabilities.

#### 3.1.6 Communication output and input

Communication output and input was coded to refer to the capabilities of the robot to share information with the users and to receive information from the users. The preferred modality of communication from users was voice. Most participants wanted the robot to speak to them and receive input from them through voice commands. For example,

*“So I think some sort of voice commands voice interaction I think that’s what people would make the most use of”* (Participant 7).

*“I do not think that Stretch has to look like a human, it just has to communicate like one”* (Participant 8).

Some compared the robot’s capability of communicating via voice and receiving information via voice commands as ‘human-like’, as they would talk to another human being in the home.

*“being able to just talk to it like you would talk to another human being and it followed all the things that she said … the fact that is doing perfectly what she was asking, this is really, really great”.* (Participant 2)

The use of the tablet interface as means of communication also inspired thoughts of human-like communication or humanness as this participant put it:

*“…And I mean, I’m impressed how well he uses an iPad like device. And that was that when we were talking earlier before and saw Stretch in action. That was very important to me that it puts human humanity into Stretch a bit. And I think that’s, that's so I think, human contact is so important that yeah, … , I’m more impressed than I was earlier.”* (Participant 5)

Some went further to express how talking to the robot would make it more personal, as this participant expressed:

“*Can the robot tell stories? People like stories. People like music, poetry. You know, person does not get out anymore…*

*… I want to I want to talk to Stretch, my robot, and we’re gonna tell scary stories to one another or something like that. And then it will bring up reminders, ‘hey, [Participant’s name], it’s time to take your medicines, you know, or it’s okay, it’s, it’s time to think about going to sleep now’. You build all kinds of personality into that top”.* (Participant 4)

### 3.2 Tasks older adults want the mobile manipulator robot to support

**FIGURE 4:**
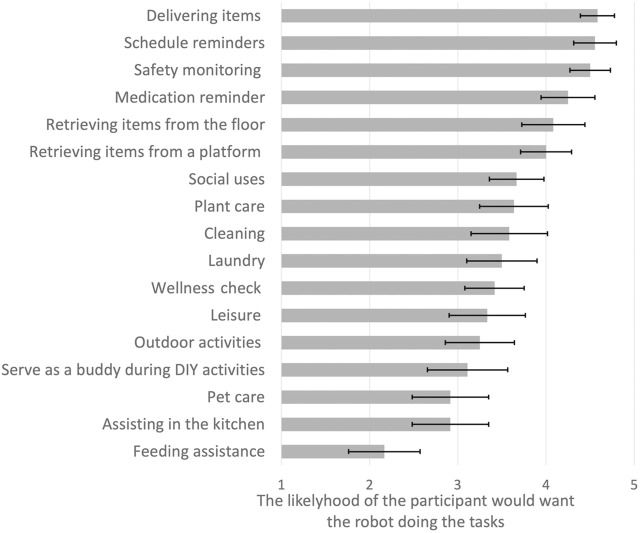
Participants’ likelihood (mean and standard error) of wanting a robot to perform various tasks (1-Not likely to 5-Very likely).

Participants mentioned cognitive support tasks including reminders for medications, healthcare appointments, and wellness checkups. They mentioned physical support tasks that involved retrieving and delivering items, cleaning, and other chores in the kitchen. Figure 4 presents a comprehensive view of the tasks participants identified as relevant to their lives. The mean system usability scale (SUS) was 73 (SD = 15.34), which reflects higher perceived usability than 70% of most products tested (Lewis, 2018).

Participants shared their overall impressions of the use of the robot for support during the interview. The top rated tasks (first 5) focus on physical support tasks (delivery and retrieval related tasks (delivering items, retrieving items from the floor), and cognitive support tasks (schedule reminders, medication reminder), and a blend of both physical and cognitive support tasks (safety monitoring–this may involve a physical task such as removing a fall risk item off the ground, and a cognitive task such as remembering to turn off the oven). Participants rated their likelihood to use the robot for these tasks as ‘very likely’ or ‘likely’.

We examined comments that the participants made as they rated these tasks. Participants with mobility impairments commented on physical tasks that they would like the robot to perform:

“*it would be useful to pick up things that are dropped, especially with my balance issues - It could be asked to retrieve something*”, “*it can help me get trip hazards off the floor*”. (Participant 2)

“*it would be very useful to help me open jars or bottle covers – tasks I cannot do now due to the condition of my hand*”. (Participant 4)

Participants with cognitive impairments shared comments related to memory support tasks:

*“medication reminders are a huge support for me”.* (Participant 6)

Most of the participants valued the potential of the robot to help in situations of a health emergency such as a fall. Most of their comments were related, as expected, to their specific health needs. They shared their thoughts not only on the tasks that would support their health but how the robot should carry out these tasks, the degree of robot autonomy that would facilitate an active and healthy lifestyle for them as well as ways they would like to control and communicate with the robot.

### 3.3 Facilitators and barriers to robot adoption by older adults

**FIGURE 5:**
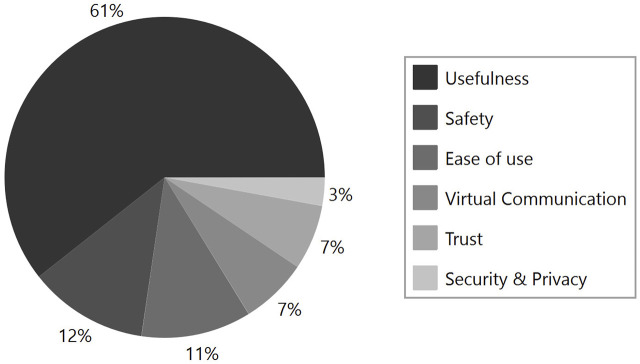
Older adults’ perceptions about factors that could impact adoption.

This section highlights some of the themes that evolved from the interview analysis after the participant had the opportunity to interact with the robot. These themes point to various aspects of the interaction that could facilitate adoption or serve as a barrier to adopting the robot. Some of these themes are presented in Figure 5. The perceived usefulness of the robot in the home environment was a major theme.

#### 3.3.1 Usefulness and ease of use

**FIGURE 6:**
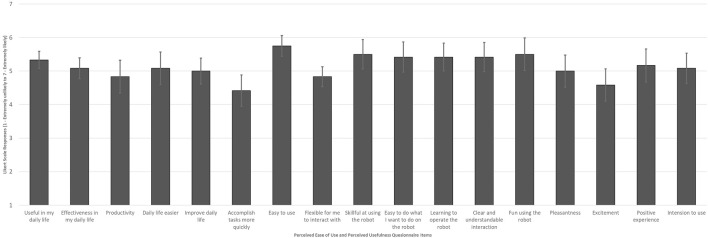
Perceived Ease of Use and Perceived Usefulness of the robot (Likert Scale 1 - Extremely unlikely to 7 - Extremely likely).

Perceived usefulness is the degree to which a participant believes that using the robot would enhance their performance. Perceived ease of use is the degree to which the participant believes that using the robot will be free of effort. The participants’ responses to the questionnaire on perceived usefulness and ease of use are presented in Figure 6. The results (M = 4.96, SD = 0.33) revealed that most participants considered the robot useful and easy to use (M = 5.33, SD = 0.24). The easy-to-use item was rated highest among all the questionnaire items.

Participants shared their overall impressions of the use of a robot in the home. Those with lower mobility impairments commented:

*“it would be useful to pick up things that are dropped, especially with my balance issues - It could be asked to retrieve something,” “It can help me get trip hazards off the floor.”* (Participant 2)

Some who had upper mobility impairments shared:

*“it would be beneficial to help me open jars or bottle covers – tasks I cannot do now due to the condition of my hand.”* (Participant 8)

Those who had cognitive impairments shared comments such as:

*“Medication reminders are a huge support for me.”* (Participant 7)

Most participants valued the robot’s potential to help in situations of a health emergency such as a fall. Most of their comments were related to their specific health needs. However, some shared thoughts of the robots helping other family members, as seen in the comment from this participant, highlighting the utility of the remote communication feature of the robot through its tablet:

*“And it’s so I’m very accustomed to this form of communicating. I communicate with my wife who is in Mexico this way, that she’s taking care of her mother who needs help. So if we could send Stretch down there, I could have my wife back. If Stretch could take care of my mother-in-law. But, so this is nice that I do not have to hold it.”* (Participant 5)

Some participants shared their thoughts not only on the tasks that would support their health but also on how the robot should carry out these tasks, the degree of robot autonomy that would facilitate an active and healthy lifestyle for them. For example,

*“It should not be doing things for you that you need to do for yourself. To be independent, you’ve got to do for yourself. And when you stop doing that, you stop functioning. You start losing a lot of strength for one thing. Right now, the thing that is difficult for me in my home is vacuuming, so I’d probably use it for that”.* (Participant 3)

#### 3.3.2 Trust

None of the participants had a drop in rating from their baseline for any of the trust items after interacting with Stretch. The participants’ willingness to trust either stayed consistent or increased by about 20% after interaction with Stretch. For example, a participant remarked,

*“it seems very reliable. Using a tablet that you know, is safe, reliable, so I can trust that and I do not see any reason not to. So that’s important. That’s good.”* (Participant 6)

*“Yeah, so I believe that Stretch could be made trustworthy.”* (Participant 11)

#### 3.3.3 Usability and workload

**FIGURE 7:**
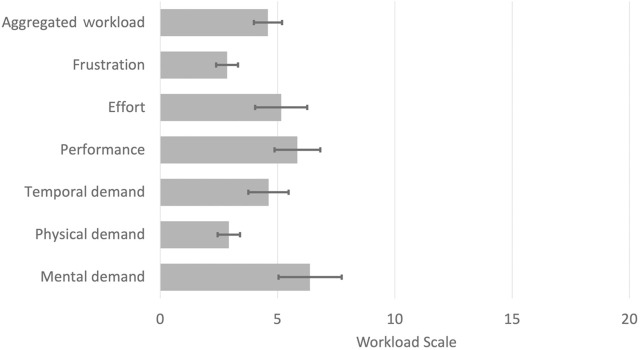
Perceptions on workload evaluated through NASA-TLX. The bar chart shows the workload ratings (from 0 to 21). Error bars represent the standard error of the mean for each dimension.

A summary of participant perceptions with regard to the workload experienced while using and interacting with the robot is presented in Figure 7. The aggregated workload (covering all workload dimensions for all the participants) pointed to a generally low workload (M = 4.59, SD = 2.16). The highest workload dimension was mental demand (M = 6.38, SD = 4.86), perhaps because of the initial learning required to get familiar with controlling the robot to execute tasks. Generally, most of the participants commented that interacting with the robot required minimal effort, which could influence their willingness to use and adopt the robot.

## 4 Discussion

### 4.1 Designing robots for older adults with mobility and/or cognitive impairments

The study provided a unique opportunity to identify the needs of older adults with mobility and cognitive impairments and the tasks the robot could perform to support these needs. There have been studies that highlighted needs of older adults that robots could support in different living environments (Mitzner et al., 2014; Beer et al., 2017; Shishehgar et al., 2019; Khosla et al., 2021; Krakovski et al., 2021; Vandemeulebroucke et al., 2021). Some have specifically identified potential of the robot to support people with cognitive impairments (Korchut et al., 2017) or mobility impairments (Ranganeni et al., 2024). Needs highlighted in those studies overlap with those that emerged from the current study. Our research thus validates some of the older adult needs identified in the literature that assistive robots could support. The current study, however, goes further to provide a more detailed understanding of specific needs that older adults with either mobility or cognitive, both, or neither of these impairments have in a typical home, observing and interacting with an assistive robot. A similar approach to understanding older adults’ acceptance of an assistive robot was conducted by Beer et al. (2017), which focused on attitudinal changes following a brief exposure. They found a set of tasks for which older adults were open, in general, for robot support. The current study adds to that by providing a more distinct understanding of specific physical support tasks, cognitive support tasks, as well as other forms of support for older adults with mobility and/or cognitive impairments. We then analyzed these tasks to identify those feasible for an assistive robot to execute. For instance, we observed commonalities in safety monitoring tasks for people with mobility and/or cognitive impairments. The robot could support both groups in this task. We also observed differences between the task preferences such as for social engagement tasks through the robot.

In the current study, we observed that integrating a social connection interface on the robot through which a caregiver, family member, or friend could communicate with the older adult as the robot performed the tasks seemed to influence the older adults’ perception and willingness to accept support from the robot. We had incorporated a tablet on the robot as the social connection interface. Participants observed Stretch’s capabilities for social connection and interacted with a research team member through the tablet interface. The video interaction feature through the robot’s tablet showed the potential to support connections with family and friends. Participants appreciated the ergonomic positioning of the tablet on the robot’s arm, making it easier for them to interact through a video call while doing other tasks. They commented on the potential ease of scheduling virtual visits and receiving reminders through the robot’s tablet for social activities. The overall impression was that this interface would facilitate better communication and social engagement and make the robot’s interaction more personal. These themes were highlighted in the systematic study conducted by Vandemeulebroucke et al. (2021) on older adults’ experiences with and perceptions of using socially assistive robots. Older adults may experience social isolation or a lack of connection with friends and family, as well as mobility and/or cognitive impairments, which significantly impact their quality of life. Designing the robot to support physical or cognitive tasks, foster social engagement, and provide emotional support is therefore beneficial. The social traits the participants mentioned that could facilitate their interaction with the robot are consistent with those mentioned in the previous studies focused on social robots (Dahl and Boulos, 2014; Ishak, 2020; Aravamuthan and Aishwarya, 2020). These include courteous responses during conversations, paying attention during interactions, and acknowledging when given instructions.

### 4.2 Gradual introduction technique for new technologies/applications

Gradual and systematic introduction of the robot to the participants seemed to ease them into the process of interacting with the robot. Most expressed increased enthusiasm and willingness to interact with the robot as they advanced through the study sections (from seeing it on the TV in the living room, watching it interact with another person, and then interacting directly with it). All the participants consented to advance to each next section of the study. We perceive, as seen in their comments and through their behavior, that this gradual introduction technique seemed to allay potential fears or anxiety of interacting with a new technology they were not familiar with. This was highlighted in the studies conducted by Naneva et al. (2020) and van Maris et al. (2020) where robot-related technophobia was found to decrease quality of engagement, substantially. These findings were further supported by a study conducted by Zafrani et al. (2023) where technophobia was identified to influence trust in the robot and as a consequence impact the older adults willingness to accept the robot. The authors advised designers to take steps to reduce concerns about robots to promote a more positive quality of engagement.

In the current study, the steps we took were to provide an understanding of the robot’s capabilities, features, and behavior through the video introduction section and then while observing the robot at a distance. We believe this provided background knowledge to the participants regarding what it could do and how they could interact with it, which seemed to provide some level of comfort and ease while interacting with the robot. Some participants initially made comments about being unsure if they could interact with the robot, but some of these comments changed to enthusiasm to further interact with the robot as they were gradually introduced to it. A similar technique was used in a study conducted by Beer et al. (2017) where pre- and post-assessments were conducted after the older adults experienced a brief exposure to the robot. The authors observed an attitudinal change, with the pre-vs.-post results indicating positive perceptions of robot usefulness and ease of use for 8 of the 12 Robot Opinions Questionnaire items. This approach provides a unique opportunity to identify changes in perception as the robot was introduced, as well as facilitators and barriers to acceptance.

### 4.3 Implications for design

This study highlights several methodological strengths, with implications for the design and deployment of robots to support older adults with a range of mobility and cognitive impairments. We demonstrated the robot’s capabilities in a realistic home environment to the participants. This was provided participants with a range of options of what the robot can do, and evoked their thoughts of what the robot should support and how it should provide the support. This was done in a systematic way with particular attention to consistency, repeatability, and relevance to the target population. We provide details of some of these insights to support designers, practitioners, and other researchers conducting similar forms of testing, participatory design, or deployment.

The home simulation environment was deliberately selected as the test environment to provide an immersive environment for participants to observe the robot in a home space and envision living in a home with a robot. This experience helped participants (as potential users) relate to potential interactions that may occur with the robot in a home space, tasks the robot could perform, and challenges inherent in the robot’s operation in a home environment. This provides a more comprehensive understanding of the peculiarities, complexities, and constraints in the user’s environment–a benefit demonstrated in the study conducted by Olatunji et al. (2024). It also helps to gain insight into some facilitators and barriers to interaction and acceptance in a home setting.

The gradual exposure of the participants to the robot was deliberate and intentional, first to ensure their safety, consent, and comfort. It provided the opportunity for the researchers to observe the attitudinal changes in the participants as they were being exposed to the robot’s potential and to identify concerns, interests, or preferences of the users as they were being introduced to the robot. For instance, if the participants were not comfortable interacting with the robot, they would have the opportunity to express that concern while they observed it in the video introduction or while interacting with one of the research team members. They could also express their thoughts in the interaction section, which was subdivided into two subsections to further encourage the participants to share thoughts on different aspects of the interaction. This three-fold gradual exposure technique provided the opportunity for the participants to see more of the capabilities and potential of the robot in a variety of ways. When discussing the benefits of trialability of a new technology (Beer et al., 2017), emphasized that the process of introducing the robot to the users greatly impacts their understanding, impressions, and likelihood of acceptance.

Researchers were in full control of the robot throughout the study. This technique ensured the safety and consistency of the robot’s actions, such as its movements, proxemic considerations, as well as the content, frequency, and mode of communication. All participants engaged in a controlled interaction, preventing any form of unplanned robot behavior. This technique enabled us to simulate various autonomous actions of the robot, and to test different forms and characteristics of engagement between the user and the robot. We were able to better study the users’ behavior and response to these carefully selected robot actions. This is one of the strategies adapted from the interaction research methods proposed by Steinfeld et al. (2009), Mitzner et al. (2014), and Rogers and Mitzner (2017) for human-robot interaction studies. It was especially ideal in our situation, where we were exploring the users’ perceptions and reactions to the robot’s interactions to improve its design.

The tasks were carefully selected and informed by literature. We aimed to demonstrate tasks that were supported by findings in previous studies and considered relevant to the target population. This provided the opportunity to showcase tasks that participants could relate to and envision trying out with the robot. It also inspired ideas in the participants for other potential tasks that the robot could support in their homes since these were relatable and relevant tasks. We recommend this approach for further studies and for designers considering a starting point for the kind of tasks to be used for demonstrating the robot’s capabilities and potential. Studies such as (Mitzner et al., 2014; Smarr et al., 2014; Wang et al., 2017; Cortellessa et al., 2021) presented a collection of potential tasks that could be used in demonstrating the robot’s potential to support older adults in various contexts.

We used a mixed methods approach in the study protocol, allowing us to address different aspects of our research questions. We were able to collect data in multiple modes such as interactive think-aloud sessions, behavioral observations through one-way mirror and overhead cameras, questionnaires, and interviews. For instance, the interactive think-aloud protocol helped users slow down as they navigated through the various sections of the study and encouraged a more participatory kind of atmosphere where their spontaneous thoughts, impressions, concerns, and ideas were valued and captured (O’Brien and Wilson, 2023). The behavioral observations through the one-way mirror and overhead cameras helped us see the participants interacting with the technology and identify changes in the distance the participants kept to the robot, their facial expressions, and overall body language during the session. This form of objective data complements the information provided through questionnaires, think-aloud data, and direct interviews, providing a more holistic understanding of the user’s attitude toward the technology and usability issues to be addressed (Lewis and Sauro, 2021).

### 4.4 Limitations of the study and future directions

One limitation of the study is our sample size (n = 12), which was relatively small but typical for a study with in-depth nature of interactions and interviews. It was, however, composed of older adults with a range of mobility and cognitive abilities. This allowed us to analyze and better understand their needs and how to design the robot to support them. The participants were independent as they were able to the venue of the study on their own and were able to independently interact with the robot. Further studies may explore the perceptions of older adults in assisted living facilities who may have a different form of needs requiring other kinds of considerations in the design. Other stakeholders (such as professional caregivers, and family care partners) may also be included in the design to get more feedback on the use of the robot to support their care recipients.

We conducted the study as a one-time visit lasting about 2.5 h. This is typically longer than most HRI studies (Bugmann and Copleston, 2011; Broadbent et al., 2012; Mast et al., 2012); however, our data may still have been affected by a novelty effect. More longitudinal studies evaluating the participants’ attitudes over time and possible changes over the course of weeks, months, or years would be of immense importance to better understand what could impact adoption.

The robot used for the study was Stretch RE2. The appearance and current limitations of the robot may have influenced user perceptions of the kind of support it could provide. It may also have influenced some of what the user expressed or did not express. For instance, seeing that the robot had only one arm and one gripper brought concerns about the robot being able to perform tasks the way we humans would with two arms. Several participants shared tasks that they wanted the robot to support if it had dual arms. However, some participants may not have suggested such tasks with the mindset that it was outside of the realm of the capability of the robot. Future work could incorporate some of the suggestions proposed by participants to further improve the robot and conduct usability testing on some of these capabilities.

Lastly, our choice of tasks, though informed by literature, could bias the participants on the kind of tasks that the robot could accomplish. We demonstrated feasible, relevant, and relatable tasks, but may have limited the boundary of possible tasks that the participants assume the robot can perform. The participants were encouraged to share their thoughts about potential tasks that the robot could support, which yielded positive results, but may still have been related in some way with what was demonstrated. Future studies could explore possibilities of the robot being in the environment of the user and the user utilizing the robot for what they would prefer to see the robot support. It may yield a different set of insights that were not observed in the more controlled tasks protocol that we adopted.

## 5 Conclusion

The goal of this study was to identify the functional capability of the robot to support older adults with a range of cognitive and mobility impairments. We engaged 12 older adults who had varying ability levels, some with cognitive and/or mobility impairments, to participate in the study to better understand their needs and to inform the design to meet these needs. We learned about the capabilities the older adults desired in the robot to support them. Prominent among these robot capabilities were autonomy, sensing capabilities, orientation and navigation, arm capabilities, and communication. The desired robot to support several tasks, with the most mentioned being delivery and retrieval tasks, as well as scheduling and medication reminders. These were physical and cognitive support tasks that cut across all the participants’ comments. The perceived usefulness of the robot was positive and related to acceptance, as were opportunities to communicate with a person (e.g., family member, caregiver, or friend) through the robot. The gradual robot exposure protocol helped older adults feel more comfortable with the new technology, minimizing unfamiliarity, anxiety, and fear. This needs assessment conducted through a participatory design approach has successfully identified ways an assistive robot can support older adults. It specifically addresses a range of cognitive and mobility impairments that older adults may have, ensuring that these individuals can actively achieve successful aging.

## Data Availability

The original contributions presented in the study are included in the article/Supplemental Material, further inquiries can be directed to the corresponding author.
