# Supplementary material for: Robotic support for older adults with cognitive and mobility impairments
Source: Front Robot AI. 2025 Apr 7;12:1545733. doi: 10.3389/frobt.2025.1545733 (PMC12010083; doi:10.3389/frobt.2025.1545733)
Supplement: Supplementary file 1 [file Table1.docx]

Supplementary Material - Robotic Support for Older Adults

with Cognitive and Mobility Impairments

# Supplementary Table

Provided below:

**Qualitative Coding Scheme**

| - General HRI Framework | - Parent Codes | - Subcodes | - Definitions | - Examples-Quotes |
| --- | --- | --- | --- | --- |
| - Human Characteristic | - Physical |  | *Physical attributes the participant mentions as it relates to what assistance they may need. The tasks that present a challenge to the participant would require assistance for due to the physical impairment. If they are speaking about another person, add the code "other".* | *“I like I have I have with my fingers I sometimes have a lot of difficulty getting my fingers to go exactly where I want them to go. So like when I'm on a keyboard, on my phone, I'm usually one letter off, because visually to where where I'm looking at my finger to be here is not where it needs to be, if that makes sense. So having that's where the reliability and robot would be really nice, because all I've got to do is remember who it is I want to call, and robots gonna take care of the rest of it.” (P7 Mobility, Pos. 291)* |
|  | - Mental Cognition |  | *Mental attributes the participant mentions it relates to what assistance they may need. The tasks that present a challenge to the participant or that they would require assistance for due to the mental ailment. If they are speaking about another person, add the code "other".* | *“Yes, safety in that sense. I know, that happens. I know, that happens with my vitamins. I know, it's like, did I take them already.” (P8 Cognitive, Pos. 523)* |
|  | - Social |  | *The participants expression of their social network, quality/quantity of connection, how they connect with others. The social tasks that present a challenge to the participantor that they would require assistance for. If they are speaking about another person, add the code "other".* | *“Because there are people who don't have physical limitations, but they've got emotional limitations. And then any autonomy the robot has could be an aide for companionship.” (P5 Mobility, Pos. 338)* |
|  | - Human Technology Experience |  | *The older adults' other life experience (e.g., background) OR What the older adult has experience in or comes to expect (with technology). The older adults' experience with technology* | *“I’m amazed at how those machines have become to get them in bed and out of bed. They didn’t have that when I was there.” (P3 Cognitive, Pos. 792)* |
| - Robot Characteristics | - Robot Capabilities |  | *The actions of Stretch (what can Stretch do, NOT task related)* |  |
|  |  | - Base Movement (Navigation) | *respect to the base, movement, how it navigates* | *“I was glad that it wasn't moving faster. Because you know, faster. Speed is not. The slower something goes, the less issues it has with interaction if it happens to bump into a pet, or whatever” (P9 Cognitive, Pos. 777)* |
|  |  | - Manipulation/ End Effector | *respect to the arm or gripper* | *“it doesn't have a laser pointer does it?” (P11 Mobility, Pos. 969-970)* |
|  |  | - Sensor Perception | *respect to the camera, sensors, environment. Between the robot and the environment.* | *“if it gave you the wrong medicine, it might even be the sorters fault. Maybe it needs to know what you're taking. Identify that medicine, just double check the dispenser.” (P5 Mobility, Pos. 385-386)* |
|  |  | - Humaness/Roboticness/Machine-like | *Comments about how the robot seems or does not seem human or machine like (not in it's appearance though), personifying Stretch. Mentioning that a task that Stretch should do is human-like* | *But I have no trouble. And I I mean, I'm impressed how well he uses an iPad like device. And that was that when we were talking earlier before […] saw stretch in action. That was very important to me that it It puts human humanity into stretch a bit. And I think that's, that's so I think, human contact is so important that yeah, no, I, I'm more impressed than I was earlier.*  *(P10 Cognitive, Pos. 1027)* |
|  | - Appearance |  |  |  |
|  |  | - Humanoid/Roboticness/Machine-like | *The physical appearance - human or robotic/machine-like* | *Uh I have mixed feelings about that I you know, you would like it to look like a kitten, or anthropomorphic or something, but it's probably better than it doesn't. It's probably better that it looks more like a tool. A mechanical device. I think that I think it might be confusing. (P11 Mobility, Pos. 162)* |
|  |  | - Size | *The physical size of Stretch including its height, width, general size, weight* | *I have to admit it just surprised me it was as tall as it is. But also have people come in different sizes to, and they're sitting on different things, or whatever. So this covers a wide range of abilities, it covers a wide range of what it can do, was a little bit of a surprise how tall it was, put in that way, but it's not a negative. Okay? (P2 Cognitive, Pos. 810-811)* |
|  | - Communication |  |  |  |
|  |  | - Input | *What or how information/cues are given to Stretch/tablet from the recipient/caregiver* | *P: Be able to talk to it. In other words, at least understand English or any other language you give it to. (P3 Cognitive, Pos. 562)* |
|  |  | - Output | *What or how information/cues are provided from Stretch/tablet to the recipient/caregiver* | *Participant 4 1:07:46*  *Can Stretch laugh?*  *Can Stretch laugh with me, you know, change the station to get a comedy station, you know, comedy [inaudibe]. It might just be really simplistic for those people that are you know, I know that a lot of elderly people, especially with dementia. They go to the past. Also Stretch should play music, because music definitely stimulates the brain and different things for a lot of people and or have that option, you know, play music, but maybe it could, I'm trying to think. I mean, you're not going to have you know, conversation in life about big life questions, you know, this isn't a psychology thing. A companion would just be something or someone that cares. Shows - you tell me how a robot shows empathy? And this is a whole big question mark here with what they are and everything. But if it's in that softer area of... (P4 Mobility, Pos. 363-365)* |
|  | - Tablet |  |  |  |
|  |  | - Type of tablet | *What tablet the participant believes Stretch should have* |  |
|  |  | - Size | *How big or small do the participants believe the current tablet is or should be* | *Oh, I suppose a little, little bigger, maybe. (P10 Cognitive, Pos. 1051)* |
|  |  | - Settings/Interface | *The various settings that can be changed for use by the older adult/caregiver* | *Speak to me unless I'm hard of hearing, in which case it needs to write to me with great big letters, because my vision might be bad, too. So can't see how big the iPad ish thing is. But it might you might need to be bigger, depending on these users. (P11 Mobility, Pos. 203)* |
|  |  | - Capabilities of the Tablet | *What the physical tablet itself should do to assist the older adult* | *Yeah. And you could probably have that voice or a voice activated keyboard or whatever where, well but they couldn't talk and they have they would be able to set it up where they could bring up somebody on their screen to communicate with you know, they couldn't talk they're if they're limited (P8 Cognitive, Pos. 499)* |
|  |  | *Robot-Autonomy* | *The degree to which Stretch should be autonomous. The ability of Stretch to do something on its own. The capacity of the robot to make its own decisions about its actions. No direct prompt/human intervention.* | *Well, that's going back to that notion. That the first thing that you do is be a companion sort of that could be really valuable to a lot of people. There are a lot of people struggling with loneliness and mental problems. That might be one of the most important things on autonomous robot could provide, this companionship. I mean, we're cutting, it's so easy to focus on mechanical tasks. Maybe it doesn't need to be. Maybe it doesn't even need to be autonomous. Maybe it just needs to be present. (P5 Mobility, Pos. 326)* |
| - Interaction | - Virtual Communication |  | *How Stretch assists with virtual interaction* | *This thing does FaceTime, I mean, I didn't get this until like four years ago, and I go, where have you been at all my life? (P12 Mobility, Pos. 930)* |
|  | - Trust |  | *Can you or can you not depend/rely on Stretch or the Hello Robot company?* | *Oh yeah. What Yeah. Stretch is a machine. Can I trust Stretch?, yeah. Can I trust stretch to break someday, Yeah, yes. You know what all my friends we're 80 plus or minus a year or two. They're kinda breaking down too. They're walking with canes and giving up their driver's license there yeah, whatever. (P10 Cognitive, Pos. 1065)* |
|  | - Adoption |  | *Would you like to have Stretch in your own home? Adoption and using it themselves in their space. If they mention their own space* | *No, not yet. My wife has Parkinson's, so it could come sooner than we know. But but not yet. The one thing that will be useful to the one thing I can think of before there's any physical disability that will be useful is medications is doesn't have to well it couldn't hurt to bring it to us but but some kind of reminder but I don't need a robot for that. I can manage that with the other electronics. (P11 Mobility, Pos. 390)* |
|  | - Usability |  | *How easy or hard is Stretch to use? Capacity of robot to provide a condition for its users to perform the tasks effectively, and efficiently while enjoying the experience. How the task is being done. Facilitators and barriers.* | *P: Well I thought, when the girl was there she asked the robot to get the basket while she was getting the groceries out. I just thought it would have been simpler for the robot to get them out and let her to get the basket because it takes longer to take the groceries out of the sack than it is to go get a basket. And the robot is supposed to be more help than another person. Another person could have gotten it and came back long before the robot. (P3 Cognitive, Pos. 782)* |
|  | *Interaction-Usefulness (tasks)* |  | *What would you like Stretch to do for you or others? How can it help the participants?* | *P: Because that would benefit me. Clothes out of the washing machine and out of the dryer. I need someone who can do the laundry who is capable, I wouldn't have to go down there. I haven’t been down there for a while because my daughter wants to get someone to do the laundry. If it weren't for the steps, I would be getting one of those acorn step things because I would just ride down the steps. (P3 Cognitive, Pos. 785)* |
|  | - Affective State |  | *What kind of emotions/bias does Stretch induce/evoke in the the older adults (if about technology in general, then just use technology experience code). STRETCH must be mentioned* | *No, I think this is my first experience with a robot, so it's a little Yeah, it's I'm, I'm fascinated. I'm impressed with what I'm impressed with what it does today. And I think next year it will do a lot more Yeah. I'm impressed with where it's going so far. And I see nothing but improvement in the future. (P10 Cognitive, Pos. 1055)* |
|  | - Safety |  | *Whether they believe Stretch or the things it can do is safe, not physically harmful. An explicit mention of harm, risk, or hazard while working with Stretch.* | *Pacticipant 8 1:05:28*  *Yes, safety in that sense. I know, that happens. I know, that happens with my vitamins. I know, it's like, did I take them already. But yeah, and I, again, with the recipe, you know, as long as you [inaudible] Stretch, I think it's the safety aspect because, again, turn off the stove when you're done. But that's sure convenient having it on there. I suppose you just, it's like Googling a recipe, and you find one, and he just lays it out for you, and tells you what to do follow the recipe. And that's pretty cool. (P8 Cognitive, Pos. 522-523)* |
|  | - Security |  | *What does the participate mention about privacy or security* | *Do any particular features make you feel uncomfortable, or unpleasant?*  *participant 11 39:26*  *No. Gotta have the camera, that would be the only thing but gotta have it so. (P11 Mobility, Pos. 220-222)* |
